# A Denture Base Resin Developed Using Hyperbranched Polyurethane Acrylate, Tricyclodecane Dimethanol Diacrylate, and Liquid Crystal Display-Based Three-Dimensional Printing

**DOI:** 10.1021/acsomega.5c08833

**Published:** 2025-12-09

**Authors:** Kuo-Chung Cheng, Cin-Yun Lin, Yu-Hsuan Chien, Yu-Hsuan Hsu, Ting-Jia Xie, Guo-Dong Hung, Mei-Wen Tseng, Bor-Shiunn Lee

**Affiliations:** † Department of Chemical Engineering and Biotechnology, 427107 National Taipei University of Technology, Taipei 106344, Taiwan; ‡ Graduate Institute of Oral Biology, School of Dentistry, 33561 National Taiwan University, Taipei 100229, Taiwan; § Department of Dentistry, National Taiwan University Hospital, Taipei 100229, Taiwan

## Abstract

Poly­(methyl methacrylate) (PMMA) is the most widely used denture base material. However, because of PMMA’s insufficient mechanical strength, fracture is a frequent problem after long-term denture use for chewing. In this study, hyperbranched polyurethane acrylate (HBPUA) was synthesized using trimethylolpropane, isophorone diisocyanate, and hydroxyethyl acrylate in a molar ratio of 1:3.1:3.2. Gel permeation chromatography revealed that the number- and weight-average weights of the synthesized HBPUA were approximately 1325 and 1797, respectively. For blended resins comprising different HBPUA and tricyclodecane dimethanol diacrylate (TCDDMDA) weight ratios, viscosity increased with increasing HBPUA content. A composition of 10 and 90 wt % HBPUA and TCDDMDA (TC10), respectively, was optimal for developing a denture base resin using liquid crystal display-based three-dimensional (3D) printing. Resins exposed to light for 5, 15, and 30 s and containing photoinitiator concentrations of 1 and 2 mol % were fabricated for comparison with a commercial resin, Denture 3D+. Attenuated total reflectance Fourier-transform infrared spectroscopy revealed that Denture 3D+ possessed a higher double bond conversion rate than those of the TC10 materials. Three-point bending tests revealed that Group 4 (TC10-0.01, 15 s) possessed the highest flexural strengths after both 50 h and 28 d of water immersion and that this group’s flexural modulus also exceeded that of Denture 3D+, although the group’s toughness and elongation were lower than those of Denture 3D+. In addition, the water sorption and solubility, surface roughness, and volumetric shrinkage of TC10 materials were lower than those of Denture 3D+. Furthermore, the impact strength, microhardness, and biocompatibility of Group 4 were comparable to those of Denture 3D+. These results indicate that Group 4 not only satisfied the ISO 20795-1 requirements but also possessed some properties superior to those of Denture 3D+, implying strong potential for practical application as a denture base material.

## Graphical abstract

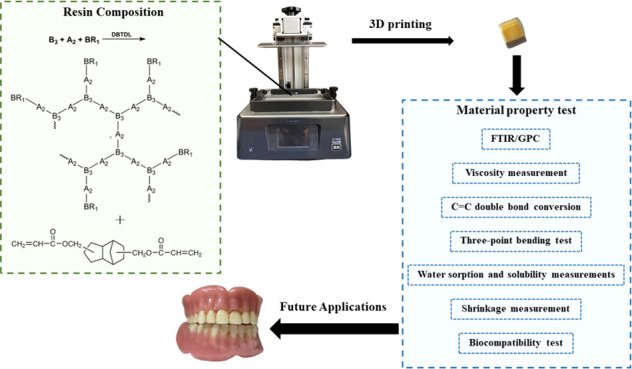

## 1 Introduction

During chewing, dentures are subjected to compressive, shear, and tensile stresses. Because of its low cost, stable color, and ease of processing, poly­(methyl methacrylate) (PMMA) is the most widely used denture base material.^1^ However, PMMA’s long-term clinical use is limited by its dimensional instability, poor mechanical strength, and vulnerability to denture base fracture.^2^ To improve PMMA’s mechanical strength, researchers have added glass or nylon fibers to PMMA, and glass fiber- reinforced PMMA possessed increased flexural strength.^3^ Although another approach has been the use of filler nanoparticles, PMMA’s hardness, flexural strength, toughness, and tensile strength were compromised by the inhomogeneous dispersion of nanoparticles in the denture polymer matrix.^4,5^ Besides the addition of fibers or nanoparticles to PMMA, chemical modification to form copolymers is another useful method to enhance PMMA’s mechanical properties. A study on the copolymerization of ethyl, butyl, and isobutyl methacrylate (IBMA) with PMMA revealed that 40% IBMA copolymerized with PMMA possessed the maximum flexural strength and modulus.^6^

Computer-aided design and manufacturing (CADM) and three-dimensional (3D) printing are recently evolved technological alternatives to conventional denture fabrication.^7^ The CADM method for milling denture resin from prepolymerized PMMA blocks can reduce labor expenditure and fabricate highly accurately fitting dentures. However, complex undercut-bearing geometries are difficult to fabricate using CADM.^8^ By contrast, in 3D printing or additive manufacturing, 3D objects are fabricated through layer-by-layer deposition.^9^ This technology can produce complex structures, reduce material waste, and is commonly used in medicine and dentistry.^10,11^ 3D printing involves various processes, including stereolithography, continuous liquid interface production, computed axial lithography, digital light processing (DLP), and remarkably high-resolution liquid crystal display (LCD)-based imaging.^12^ When an electric field is applied to liquid crystals, their molecular alignment changes, thereby blocking light passage. However, LCDs possess a relatively short lifespan and require regular replacement. In addition, in LCD-based 3D printing, the light intensity is very low, as only approximately 10% of the light can pass through the LCD screen, with approximately 90% being absorbed. Despite these drawbacks, LCD printers are a highly affordable and feasible option in certain dental applications, such as occlusal splints and gingiva masks for implant models.^13,14^ DLP is another widely utilized 3D printing process capable of fabricating aesthetic zirconia dental crowns with translucency and color gradation,^15^ orthodontic aligner attachments,^16^ and resin-based dental provisional crowns and bridges.^17^

Instead of the traditional processing of PMMA denture bases, researchers have employed PMMA-based 3D printing. However, PMMA’s clinical applications are limited by insufficient mechanical strength and a high shrinkage rate during light- induced polymerization.^18^ Because of their high viscosities, other commonly used dental resins, such as bisphenol A-glycidyl methacrylate (Bis-GMA) and urethane dimethacrylate, are also difficult to print.^19^ A previous study investigated the mechanical properties (flexural strength and modulus, Vickers hardness, and surface roughness) of two commercial 3D-printed denture base resins (Detax and NextDent 3D+) incorporating 3% w/v of powdered essential oil microcapsule,^20^ and the results revealed that although the hardness and flexural modulus remained relatively unchanged, the powdered microcapsules decreased and increased the flexural strength and surface roughness, respectively. Another study evaluated the effects of ZrO_2_ nanoparticles on the flexural and impact strengths, hardness, surface roughness, and elastic modulus of commercial 3D-printed NextDent and ASIGA denture base resins,^21^ and the results revealed that the ZrO_2_ nanoparticles substantially increased the flexural and impact strengths and hardness but negligibly affected the surface roughness and elastic modulus.

Hyperbranched polymers (HBPs) possess unique structures, where the polymer chains branch out in a dendritic-like pattern, hindering their entanglement.^22^ HBP viscosities are lower than those of similar molecular weight linear polymers. Polyurethane (PU) possesses favorable mechanical properties, biocompatibility, and a wide range of practical applications. Combined with functional groups, such as acrylate, PU is used to prepare ultraviolet (UV)- or visible light-curable resins photopolymerized at room temperature.^23,24^ In our previous work, hyperbranched polyurethane acrylate (HBPUA) was prepared using A_2_/B_3_/BR-type stepwise polymerization in a batch reactor,^25–27^ and the results revealed that the microhardness, flexural strength and modulus, shrinkage, and biocompatibility of the photocured resin prepared using 60 and 40 wt % HBPUA and triethylene glycol dimethacrylate (TEGDMA) were either comparable or superior to those of the commercial dental resin Luxatemp (DMG, Warrington, UK) that the blended resin could be practically applied to provisional dental prostheses, and that HBPUA was an ideal material for LCD-based 3D printing.

Tricyclodecane dimethanol diacrylate (TCDDMDA) is a cycloaliphatic monomer possessing a tricyclic central group and difunctional ends and is used as a cross-linking agent. The TCDDMDA pendant acrylate group is highly reactive and easily polymerizes with other carbon–carbon double bonds. A previous study has shown that methyl methacrylate (MMA) containing 20% TCDDMDA possessed the highest flexural and impact strengths for denture bases.^28^ Moreover, teeth fabricated using a thermally aged and cyclically loaded copolymer resin comprising PMMA and 20% TCDDMDA also possessed higher shear bond strength^29^ and possessed favorable histocompatibility in rats, exhibiting no cytotoxicity toward murine fibroblasts.^30,31^

In this study, HBPUA was prepared via A_2_/B_3_/BR-type stepwise polymerization and then blended with TCDDMDA. A commercial 3D-printed denture base resin, Denture 3D+ (NextDent, Soesterberg, Netherlands) was used for comparison. Subsequently, LCD was used to fabricate resin specimens. The water sorption and solubility, volumetric shrinkage, impact strength, microhardness, surface roughness, and mechanical properties (including the flexural strength and modulus, toughness, and elongation after water storage for both 50 h and 28 d) of the resins were measured. In addition, the biocompatibilities of the resins were investigated using cell-counting kit-8 (CCK-8) assays.

## 2 Experimental Section

### 2.1 Materials

Trimethylolpropane (TMP) and dibutyltin dilaurate (DBTDL) were purchased from Sigma-Aldrich, St. Louis, MO, USA. Isophorone diisocyanate (IPDI) was obtained from Acros Organics, Geel, Belgium. Hydroxyethyl acrylate (HEA) was purchased from Tokyo Chemical Industry, Tokyo, Japan. TCDDMDA was purchased from ACT Chemical Corp., Taipei, Taiwan. The photoinitiator 2,4,6-trimethylbenzoyl phosphine oxide (TPO) was purchased from Chembridge International Corp., Taipei, Taiwan. All the chemicals were used as received without further purification.

### 2.2 Synthesis of HBPUA

The synthesis of HBPUA has been described previously.^27^ A trifunctional monomer TMP (B_3_), a difunctional monomer IPDI (A_2_), and an end-capping compound HEA (BR) were dissolved separately and uniformly in anhydrous acetone in a molar ratio of 1:3.1:3.2. Once completely dissolved, the solutions were sequentially added to a three-neck flask and stirred thoroughly to ensure homogeneous mixing. Subsequently, the DBTDL catalyst was introduced to immediately initiate the reaction. The experiment was conducted under anhydrous and oxygen-free conditions in a nitrogen atmosphere at 46 °C for 10 h. The system’s cooling medium was maintained at 4 °C to decelerate acetone evaporation. Upon completion of the reaction, acetone was removed via vacuum distillation at 42 °C, producing HBPUA. Figure 1 shows the chemical structures of HBPUA, TMP, IPDI, HEA, and TCDDMDA.

**Figure 1:**
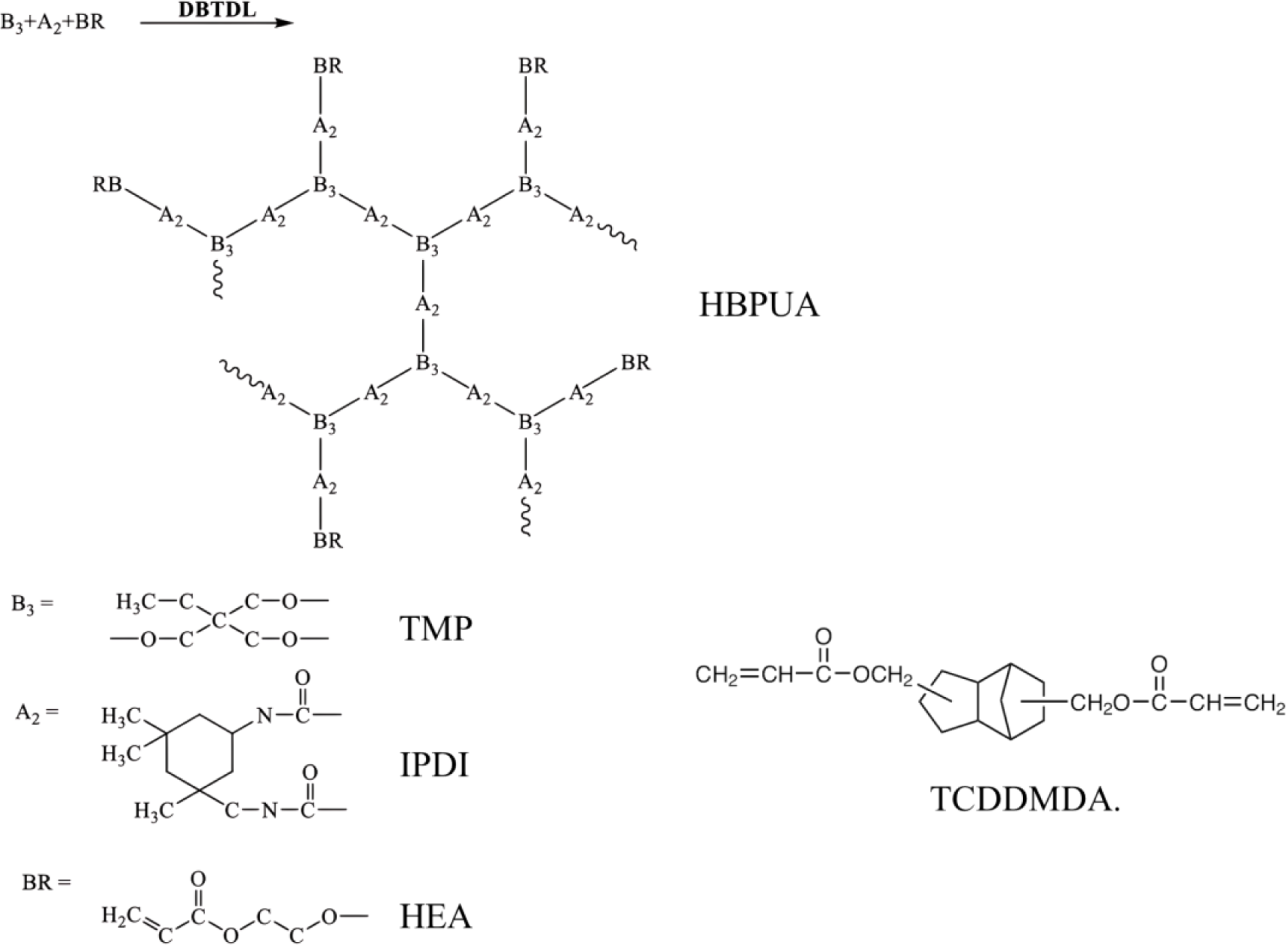
Chemical structures of HBPUA, TMP, IPDI, HEA, and TCDDMDA.

### 2.3 Attenuated Total Reflectance Fourier-Transform Infrared (ATR-FTIR) Spectroscopy

During HBPUA synthesis, the reactive functional group isocyanate (−NCO) conversion rate was quantified using ATR-FTIR spectroscopy (Spectrum 3, PerkinElmer, Shelton, USA). The spectral range was set from 4000 to 450 cm^–1^ at a resolution of 4 cm^–1^, and the conversion rate was determined based on changes in the intensities of two characteristic peaks for the reactants at T0 (completely dissolved in acetone and homogeneously mixed for 1 min) and after HBPUA formation (peaks corresponding to the isocyanate (−NCO) functional group and alkyl (C–H) reference at 2267 and 2954 cm^–1^, respectively). The conversion rate was calculated using eq 1

1 $$\alpha_{\mathrm{isocyanate}}=1-\frac{A_{-\mathrm{NCO}}^{-t}/A_{\mathrm{ref}}^{-t}}{A_{-\mathrm{NCO}}^{-0}/A_{\mathrm{ref}}^{-0}}$$

where α_isocyanate_ represents the isocyanate conversion rate, *A* denotes the absorbance, and *A* ^–0^ and *A*–^*t*^ refer to the absorbance values for the reactants at T0 and after HBPUA formation, respectively.

### 2.4 Determination of the Number- and Weight-Average Weights of the Synthesized HBPUA Using Gel Permeation Chromatography (GPC)

A series of linear polystyrene standards possessing molecular weights of 34800, 9630, and 3220 g mol^–1^ was used as calibration standards, along with an IPDI- and HEA-derived synthesized trimer as a known low-molecular weight standard. Tetrahydrofuran (THF) was employed as the mobile phase, flowing at 0.5 mL min^–1^, and the column temperature was maintained at 40 °C. Samples were prepared by dissolving the polymer in THF in a weight ratio of 1:30 and ultrasonically agitated until a clear, particle-free solution was obtained. The solutions were then filtered 4–5 times using a syringe filter (13 mm in diameter, 0.22 μm pore, and a hydrophobic polytetrafluoroethylene (PTFE) membrane) to ensure complete clarification. The filtered samples were subsequently injected into a GPC system to determine the molecular weight and distribution of the HBPUA. GPC was equipped with a refractive index detector (RI-2000, Schambeck, Bad Honnef, Germany) and two chromatographic columns (Shodex, Tokyo, Japan): SHODEX KF-804L (300 × 8 mm) and SHODEX KF-802L (300 × 8 mm).

### 2.5 Viscosity Measurements

The viscosities of the blended resins comprising HBPUA (0, 5, 10, 15, or 20 wt %) and TCDDMDA (100, 95, 90, 85, or 80 wt %) in 1 mol % of TPO photoinitiator were measured using a rheometer (MCR 302, Anton Paar, Graz, Austria) at 15, 20, 25, 30, and 37 °C. A temperature controller was connected to a water bath, and the viscosities were measured in the shear rate range from 0.01 to 200 s^–1^.

### 2.6 3D-Printed Specimen Preparation

An LCD printer (Sonic Mini 8K, Phrozen, Hsinchu, Taiwan) and ChiTuBox64 V 1.9.0 software were used. The printer uses light with a 405 nm wavelength, 22 μm (1152 ppi) resolution, and a printing space with an 18 cm *Z*-axis. Eight groups (Table 1) were included in this study. Group 1 used a 3D-printed commercial denture base resin (Denture 3D+), where each printing layer was 0.05 mm high, and the resin was exposed to light for 5 s. Group 1 was denoted as Denture 3D+, 5 s. Group 2 also comprised Denture 3D+, where each printing layer was 0.03 mm high, and the resin was exposed to light for 15 s. Group 2 was denoted as Denture 3D+, 15 s. Groups 3–8 comprised HBPUA and TCDDMDA mixed in a weight ratio of 10:90, and the printing layer height was set at 0.03 mm. Photoinitiator TPO was added at 1 and 2 mol % to Groups 3–5 and 6–8, respectively. The light exposure times of Groups 3–8 were 5, 15, 30, 5, 15, and 30 s, respectively. Therefore, Groups 3–8 were denoted as TC10-0.01, 5 s; TC10-0.01, 15 s; TC10-0.01, 30 s; TC10-0.02, 5 s; TC10-0.02, 15 s; and TC10-0.02, 30 s, respectively, as shown in Table 1. After the resins were printed, 95% ethanol was used to clean the residual resin monomer on the surface of the 3D-printed specimens for 3 min. All the specimens were postcured under UV irradiation (Form Cure, Formlabs, Somerville, USA) at 60 °C for 30 min.

**Table 1:** Groups and Printing Parameters of 3D-Printed Specimens

Groups	Exposure time (s)	Layer height (mm)
Group 1: Denture 3D+, 5 s	5	0.05
Group 2: Denture 3D+, 15 s	15	0.03
Group 3: TC10-0.01, 5 s	5	0.03
Group 4: TC10-0.01, 15 s	15	0.03
Group 5: TC10-0.01, 30 s	30	0.03
Group 6: TC10-0.02, 5 s	5	0.03
Group 7: TC10-0.02, 15 s	15	0.03
Group 8: TC10-0.02, 30 s	30	0.03

### 2.7 Calculation of C=C Double Bond Conversion (DBC) Rates

ATR-FTIR spectroscopy was used to analyze the resin samples before and after 3D printing. Disk-shaped specimens (15 mm in diameter and 1 mm thick) were prepared for measurements in the range from 4000 to 450 cm^–1^ at a resolution of 4 cm^–1^. Changes in two characteristic peaks—the C=C and C=O stretching vibrations at 1636 and 1720 cm^–1^ (representing the acrylate double bond and used as an internal reference, respectively)—were analyzed to calculate the DBC rate. The acrylate DBC rate was calculated using eq 2 (*n* = 6):

2 $$\mathrm{DBC rate}\;\left(\%\right)=\left[1-\frac{A_{C=C1636{\mathrm{cm}}^{-1}}/A_{C=O1720{\mathrm{cm}}^{-1}}\;\left(\mathrm{polymerized}\right)}{A_{C=C1636{\mathrm{cm}}^{-1}}/A_{C=O1720{\mathrm{cm}}^{-1}}\;\left(\mathrm{resin}\right)}\right]\times 100\%$$

### 2.8 Three-Point Bending Tests

This experiment followed the procedures specified in the International Organization for Standardization’s ISO 20795-1:2013 standard for denture base polymers. Specimens were printed as 64 mm long, 10 ± 0.2 mm wide, and 3.3 ± 0.2 mm high rectangular bars and immersed in water at 37 °C for 50 ± 2 h prior to testing. In addition, to evaluate the long-term stability of the materials, the other specimen groups were immersed in water at 37 °C for 28 d to simulate extended clinical water exposure.

Subsequently, each specimen was placed on a three-point bending fixture at a support span of 50 ± 0.1 mm and tested in a water bath maintained at 37 °C. The test was performed using a universal testing machine (YM-H3501-A02, Yang Yi Technology Co., Ltd., Tainan, Taiwan), applying a downward displacement at 5 ± 1 mm min^–1^ until the specimen fractured. The maximum load and corresponding displacement at the maximum load were recorded and used to calculate the flexural strength and modulus of the samples (*n* = 6). The calculation formulas are shown as eq 3 and 4.

3 $$\mathrm{Flexural strength}=\frac{3Fl}{2bh^{2}}$$

4 $$\mathrm{Flexural modulus}=\frac{3F_{1}l^{3}}{2bh^{3}d}$$

where *F* is the maximum load applied to the specimen (N), *l* is the length of the support span (50 ± 0.1 mm), *b* is the width of the specimen (10 ± 0.2 mm), *h* is the height (thickness) of the specimen (3.3 ± 0.2 mm), *F*_1_ is the load at a selected point on the linear portion of the load–deflection curve (maximum slope (N)), and *d* is the deflection at load *F*_1_ (mm).

In addition, toughness, defined as the area under the stress–strain curve, representing the total energy absorbed before fracture, was also determined. Elongation at break, referring to the displacement at the breaking point relative to the original span, was measured to assess the material’s flexibility and ductility.

### 2.9 Impact Strength

The Izod impact strength of the printed specimen (64 mm in length × 12.7 mm in width × 3.2 mm in thickness) (*n* = 6), in accordance with the ASTM D256 specification, was measured using a pendulum impact tester (GT-7045-MDL, Gotech, Taichung, Taiwan). A V-shaped notch (tip radius of 0.25 mm, depth of 2 mm, angle of 45°) was created in the middle of the specimen (Figure 2). The pendulum swung, fell, and quickly struck the specimen, which was fixed in a groove. The Izod impact test indicates the energy required to break the notched specimen under standard conditions.

**Figure 2:**
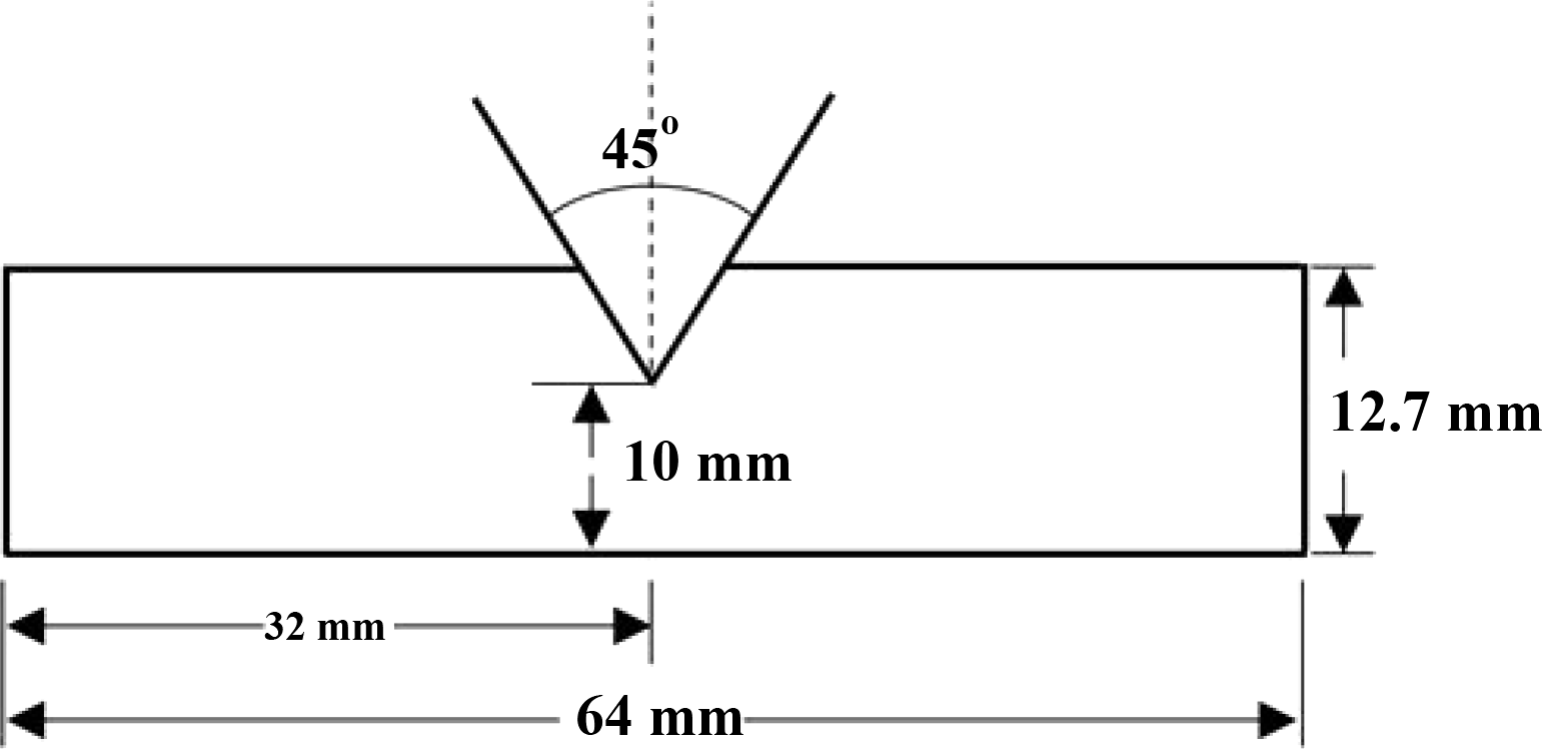
Printed specimen (64 mm in length × 12.7 mm in width × 3.2 mm in thickness), featuring a V-shaped notch (tip radius of 0.25 mm, depth of 2 mm, and an angle of 45°) was used for the Izod impact strength measurement, with the notch created in the middle of the specimen.

### 2.10 Microhardness Measurement

Cubic specimens (5 × 5 × 5 mm) were used (*n* = 6). After the resins were printed, two indentations were made on the top surface of each specimen using a microhardness tester (HMV-2, Shimadzu, Kyoto, Japan), and the average Vickers hardness number (VHN) was calculated. A diamond indenter with a load of 0.98 N and a dwell time of 15 s was used to make indentations.

### 2.11 Surface Roughness Measurement

Specimens (*n* = 3) were prepared as circular discs with a diameter of 50 ± 1 mm and a thickness of 0.5 ± 0.1 mm. Each specimen was ground and polished for 3 min using a Buehler Ecomet 3 grinder/polisher (Buehler Ltd., Lake Bluff, IL, USA) equipped with 4000-grit silicon carbide waterproof abrasive paper under continuous water cooling to achieve a uniform and smooth surface prior to testing. Surface roughness was measured using a surface profilometer (Surfcorder ET 200, Kosaka, Tokyo, Japan). The tracing diamond tip had a radius of 2 μm, with a tracing speed of 0.2 m/s, a force of 200 μN, a tracing length of 4 mm, and a cutoff value of 0.8 mm. Five tracings were performed at different locations on the surfaces of each specimen. The average surface roughness (Ra) values of the three specimens in each group were calculated.

### 2.12 Shrinkage Measurements

The shrinkage rate was determined by measuring the change in the specific gravity of the resin before and after printing. Cubic specimens (5 × 5 × 5 mm) were used (*n* = 6). Before printing, the liquid density was measured using the gravimetric method. A 10 mL volumetric flask was weighed empty and then reweighed after being filled with liquid resin. The mass difference corresponded to the resin’s mass, which was then divided by the density of water (1 g cm^–3^) to calculate the specific gravity of the liquid resin.

After the resins were printed, the solid densities were measured using a digital analytical balance (AP225WD, Shimadzu Corporation, Kyoto, Japan) equipped with a density determination kit (SMK-401, Shimadzu Corporation, Kyoto, Japan). According to Archimedes’ principle, the buoyant force on an object submerged in a liquid equals the weight of the displaced liquid. Specimens were first weighed in air and then weighed while submerged under water. The difference corresponded to the weight of the displaced water, which, because the density of water is 1 g cm^–3^, is numerically equal to the specimen’s volume. The specific gravity of the solid was then calculated by dividing the specimen’s weight in air by its volume.

The shrinkage rate was calculated using eq 5.

5 $$\mathrm{Shrinkage}\;\left(\%\right)=\frac{\rho_{2}-\rho_{1}}{\rho_{2}}$$

where ρ_2_ and ρ_1_ are the specific gravities of the printed solid and liquid (before printing), respectively.

### 2.13 Water Sorption and Solubility Measurements

This experiment was also conducted according to procedures specified in the ISO 20795-1:2013 standard to evaluate the water sorption and solubility (*W*sp and *W*sl, respectively) of the materials.

Specimens (*n* = 6) were prepared as circular discs (50 ± 1 mm in diameter and 0.5 ± 0.1 mm thick). The diameter and thickness of each specimen were measured three times at random locations, and the average values were used to calculate and record the specimen volume (*V*). Each specimen was first dried in a 37 °C oven for 24 h to remove moisture, and the dried specimen weight was recorded as *m*_1_. The dry specimen was then immersed in water at 37 °C for 7 d, after which the weight was recorded as *m*_2_. Following immersion, the specimen was redried in the oven at 37 °C for another 24 h, and the final weight was recorded as *m*_3_.

*W*sp and *W*sl were calculated using eqs 6 and 7.

6 $$W\mathrm{sp}=\frac{m_{2}-m_{3}}{V}$$

7 $$W\mathrm{sl}=\frac{m_{1}-m_{3}}{V}$$

### 2.14 Material Biocompatibilities

The human gingival fibroblast cell line HGF-1 (ATCC CRL-2014) was obtained from the American Type Culture Collection (ATCC; Manassas, VA, USA). The cells were cultured in Dulbecco’s Modified Eagle’s Medium supplemented with 10% (v/v) fetal bovine serum and 1% penicillin–streptomycin. Cultures were maintained at 37 °C in a humidified incubator with 5% CO_2_. The cytotoxicities of Groups 2, 4, and 7 materials was evaluated using HGF-1 cells according to ISO 10993 guidelines for biologically evaluating medical devices. The assays were conducted following the procedures specified in the ISO 10993-5 standard. In compliance with the ISO 10993–12 standard, specimens (>0.1 mm thick) were extracted using a standardized surface area-to-extraction volumetric ratio of 3 ± 10% cm^2^ mL^–1^. Extraction was performed by incubating the samples (20 mm in diameter and 2 mm thick) in complete culture medium at 37 °C for 72 h to obtain the extract solutions.

HGF-1 cells were seeded into 96-well plates at 5 × 10^3^ cells per well and incubated for 24 h to facilitate cell attachment. The culture medium was then replaced with either the material extract or fresh medium (in the experimental and control groups, respectively) and then incubated for an additional 24, 48, or 72 h. At each time point, 10 μL of CCK-8 (Sigma, St. Louis, MO, USA) solution was added to each well, and the cells were incubated for 2 h. The optical densities were measured using a microplate photometer at 450 nm (Multiskan FC; Thermo Fisher Scientific, Waltham, MA, USA). Relative cell viabilities were calculated by normalizing absorbance values to that of the control group (set at 100%). A cell viability exceeding 70% was considered as indicative of noncytotoxicity.

### 2.15 Statistical Analysis

A one-way analysis of variance was performed using Statistical Package for the Social Sciences software (v. 22.0, IBM, Armonk, NY, USA) to analyze the differences between values. The significance level was 0.05 based on Tukey–Kramer multiple comparison tests.

## 3 Results and Discussion

### 3.1 FTIR Analysis

Figure 3 shows the FTIR spectra of the reactants at T0, HBPUA, TMP, IPDI, and HEA. Some prominent peaks were identified at T0 (Figure 3a), including a broad absorption band at 3400 cm^–1^, corresponding to the stretching vibration of the hydroxyl (−OH) group;^32^ a peak at 2954 cm^–1^, corresponding to stretching vibrations of C–H, which did not participate in the reaction;^32^ a strong and sharp peak at 2267 cm^–1^, corresponding to the isocyanate (N=C=O) group;^33^ and peaks at 1720, 1636, and 1235 cm^–1^, corresponding to C=O,^34^ C=C,^34^ and C–O^32^ stretching vibrations, respectively. After the reaction between the isocyanate and hydroxyl groups, HBPUA’s urethane linkages formed, and the peak at 2267 cm^–1^, corresponding to isocyanate, prominently weakened (Figure 3b), indicating N=C=O group consumption. In addition, a peak at 1530 cm^–1^, representing C–N stretching and −NH bending vibrations, confirmed urethane bond formation.^35^ The FTIR spectrum of TMP exhibited a prominent hydroxyl (−OH) absorption peak (Figure 3c). The peak at 1475 cm^–1^ corresponded to C–H bonds’ in-plane bending, and the band at 1346 cm^–1^ was attributed to the symmetric bending vibration of C–H bonds in methyl (CH_3_) groups.^36^ The FTIR spectrum of IPDI exhibited a characteristic absorption peak at 2267 cm^–1^ for the isocyanate (N=C=O) group and did not exhibit any signals corresponding to the hydroxyl (−OH) group (Figure 3d). The FTIR spectrum for HEA exhibited a distinct C=C absorption peak at 1636 cm^–1^, a peak corresponding to hydroxyl group (−OH) stretching at 3400 cm^–1^, and a band corresponding to C=O (ester) stretching at 1720 cm^–1^, indicating ester functional groups in the acrylate monomer (Figure 3e).

**Figure 3:**
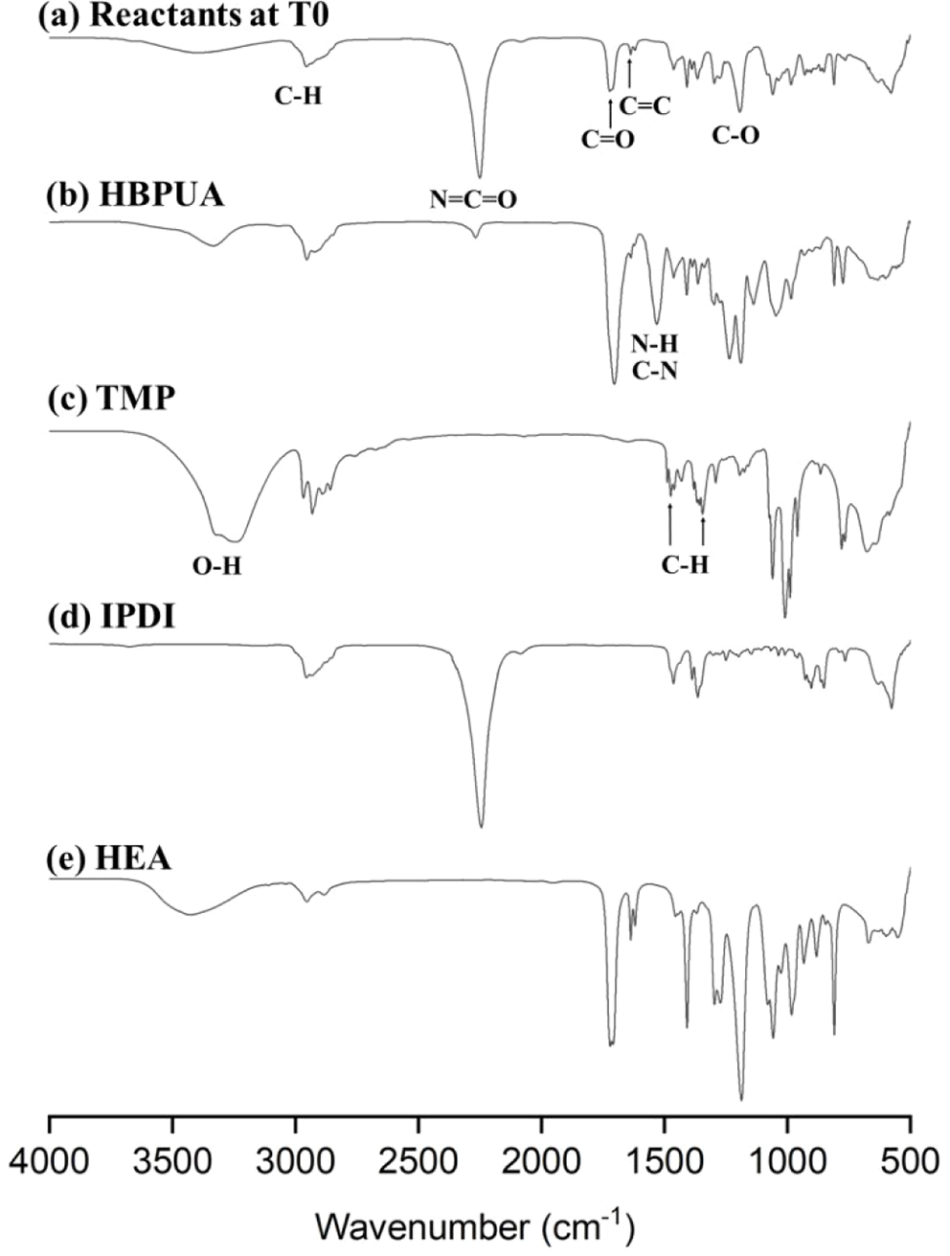
FT-IR spectra of (a) reactants at T0, (b) HBPUA, (c) TMP, (d) IPDI, and (e) HEA. T0 was defined as all the reactants being completely dissolved in acetone and homogeneously mixed for 1 min.

### 3.2 Number- and Weight-Average Weights of HBPUA and Urethane Diacrylate

Figure 4a shows the GPC chromatograms of the polystyrene standard used to establish the molecular weight calibration curve. HBPUA possessed the highest molecular weight and a broad molecular weight distribution (Figure 4b), indicating a certain degree of polydispersity. Notably, the GPC trace of HBPUA also exhibited a peak in the lower-molecular weight region, which was attributed to the formation of urethane diacrylate (Figure 4c)—a linear byproduct generated from the reaction of IPDI with two molecules of HEA during synthesis. Urethane diacrylate lacks reactive terminal functional groups that can further react with TMP to form a cross-linked or branched structure. Therefore, the peak corresponding to urethane diacrylate appeared in a lower-molecular weight region in the GPC chromatogram.

**Figure 4:**
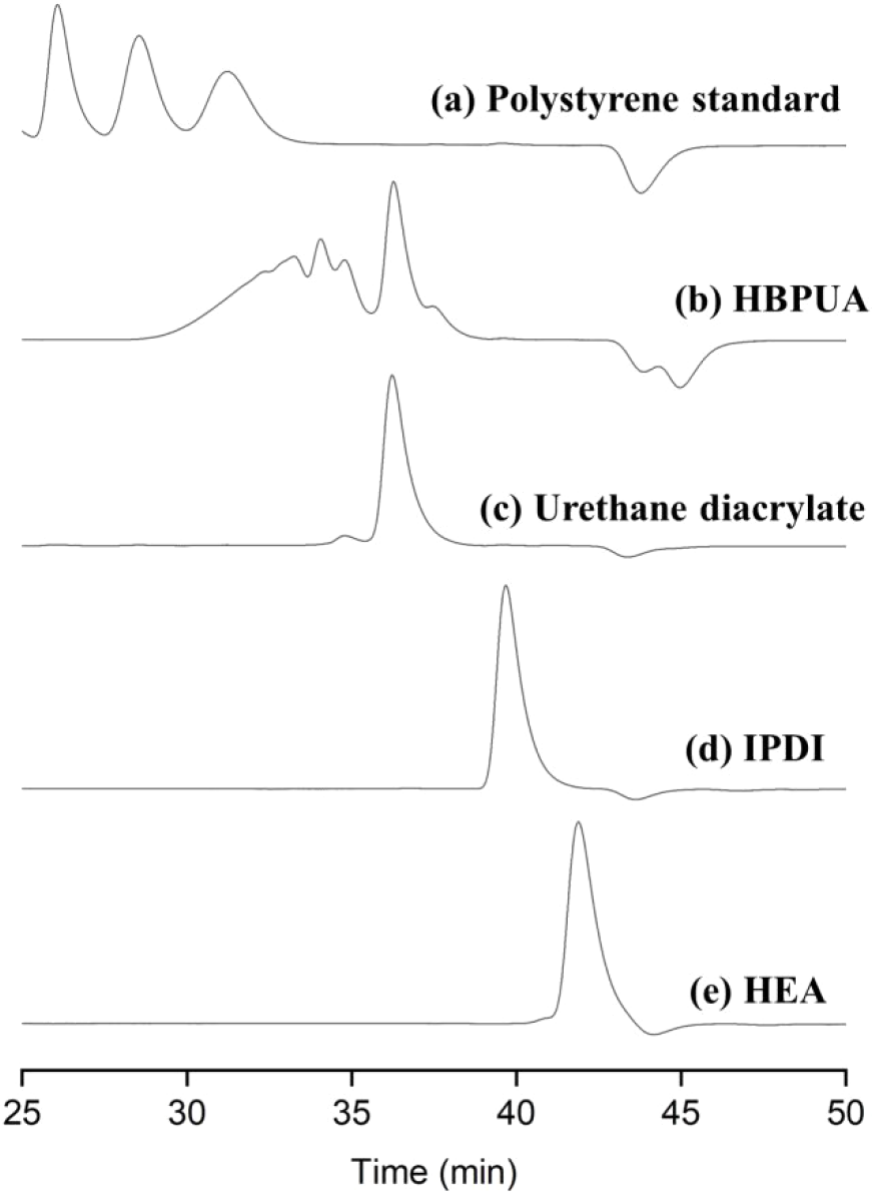
GPC chromatograms of (a) polystyrene standard, (b) HBPUA, (c) urethane diacrylate, (d) IPDI, and (e) HEA.

As shown in Figure 4, the HBPUA chromatogram did not exhibit any characteristic peaks corresponding to IPDI or HEA monomers, indicating that the monomers fully participated in the reaction. Consequently, the −NCO functional group signal in the HBPUA chromatogram (Figure 3) was not attributed to residual unreacted IPDI monomers. Because each IPDI molecule contains two −NCO groups, the detected −NCO signal was more likely attributable to partially reacted IPDI units, where only one −NCO group reacted with an −OH group, while the other −NCO group did not react, suggesting that monofunctional IPDI segments contributed to the residual isocyanate absorption band.

The weight- and number-average weights (*M*_w_ and *M*_n_) of the synthesized HBPUA, as determined using GPC, were approximately 1797 and 1325, respectively (Table 2). In addition, for urethane diacrylate (Figure 5), comprising IPDI and HEA, Mw and Mn were 430 and 410, respectively. The isocyanate conversion rates (α_isocyanate_) of HBPUA and urethane diacrylate were 94% and 96%, respectively.

**Table 2:** Compositional Molar Ratios and Number- and Weight-Average Weights of HBPUA and Urethane Diacrylate

	Composition (molar ratio)					
	B_3_	A_2_	BR			
Sample	TMP	IPDI	HEA	*M*_w_	*M*_n_	α _Isocyanate_ (%)
HBPUA	1	3.1	3.2	1797	1325	94
Urethane diacrylate	0	1	2	430	410	96

**Figure 5:**
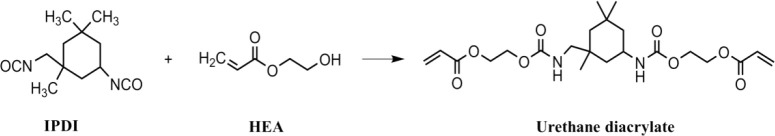
Schematic for urethane diacrylate synthesis.

During HBPUA synthesis, both HEA and TMP contained a hydroxyl (−OH) functional group that could react with isocyanate (−NCO) groups in IPDI. Therefore, HEA and TMP randomly reacted with −NCO groups in IPDI. Because HEA possesses only a single hydroxyl group, when both −NCO groups in an IPDI molecule react with HEA, the product possesses a symmetrical structure and cannot further participate in polymerization, forming urethane diacrylate. Therefore, HBPUA contained a certain proportion of urethane diacrylate.

### 3.3 Shear Viscosity Measurements

For all the materials, shear viscosity decreased with increasing temperature (Table 3). In addition, the viscosity increased with increasing HBPUA content. Although the molecular weight of the TCDDMDA monomer was 304.4 g mol^–1^, Mn and Mw of HBPUA were approximately 1325 and 1797, respectively, suggesting that the higher-molecular weight-bearing HBPUA increased the shear viscosity. A previous study has suggested that the viscosities of 3D-printing resin mixtures should be lower than 1.5 Pa·s to avoid voids or missing layers.^18^ During 3D printing, higher contents of higher-viscosity HBPUA (e.g., T15 and T20) could produce a nonuniform structure, negatively affecting the sample’s mechanical properties. In addition, because the environmental temperature was approximately 25–30 °C during 3D printing, TC10 materials (possessing higher mechanical strengths) were chosen to facilitate printing.

**Table 3:** Composition and Shear Viscosities of Materials Tested at Different Temperatures

	Composition (weight ratio%)		Shear rate = 1 (1/s), Viscosity (Pa·s)				
Codes	HBPUA	TCDDMDA	15 °C	20 °C	25 °C	30 °C	37 °C
TC0	0	100	0.32	0.22	0.15	0.10	0.06
TC5	5	95	0.42	0.28	0.19	0.13	0.08
TC10	10	90	0.63	0.40	0.25	0.17	0.11
TC15	15	85	1.12	0.70	0.45	0.30	0.18
TC20	20	80	2.13	1.39	0.81	0.53	0.31

TCDDMDA is a cycloaliphatic monomer used as a cross-linker and possesses both a central tricyclic ring group and steric hindrance, reducing and increasing the polymerization and monomer-to-polymer conversion rates, respectively.^28^ Compared with traditional PMMA-based denture base resin, TCDDMDA possesses a highly reactive pendant acrylate group and difunctional dual reactive carbon–carbon double bonds that facilitate faster polymerization. HBPUA possesses abundant carbon–carbon double bonds; thus, the photocuring rate of the HBPUA-TCDDMDA polymer blend increased.

### 3.4 Calculation of C=C DBC Rate

The C=C DBC rate (%) was determined using FTIR spectroscopy to observe the shifts in peaks corresponding to C=C and C=O stretching vibrations at 1636 and 1720 cm^–1^, respectively, before and after resin polymerization. Figure 6a shows representative FTIR spectra of the Denture 3D+ resin before and after polymerization. After polymerization, the C=C stretching vibration at 1636 cm^–1^ notably weakened. Figure 6b shows the C=C DBC rates of the printed resins exposed to light for various lengths of time. Overall, within the same material group, the C=C DBC rate increased with prolonged light exposure. Group 2 possessed the highest DBC rate, which was significantly higher than those in Groups 4 and 7. Moreover, in both Groups 1 and 2, the DBC rates were significantly higher than those in Groups 3 and 6. However, in Groups 6–8 the DBC rates were not higher than those in Groups 3–5 for the same light exposure times, suggesting that the conversion might have been saturated and that further increasing the TPO concentration negligibly enhanced the measured DBC rates.

**Figure 6:**
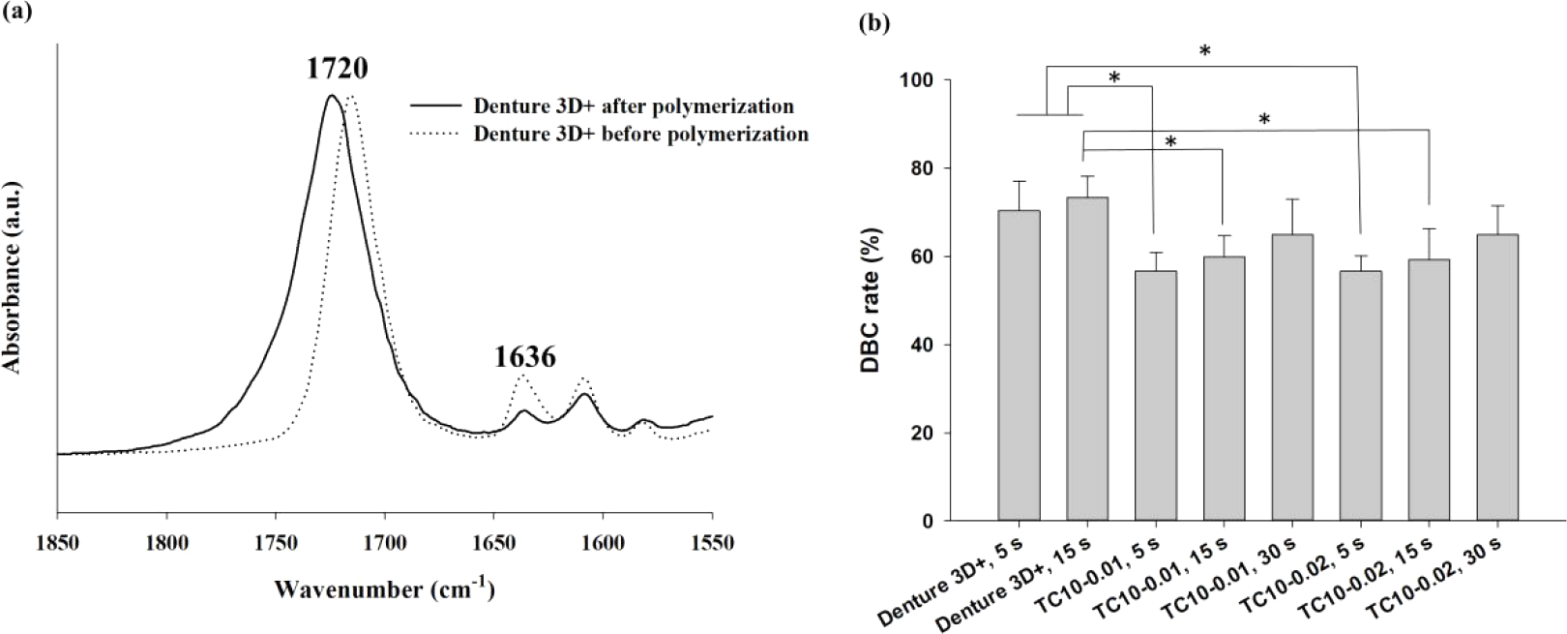
(a) Representative FTIR spectra of Denture 3D+ resin before and after polymerization, showing a weakened C=C stretching peak (at 1636 cm^–1^). (b) C=C DBC rates (%) of Denture 3D+ and TC10 resins exposed to light for various lengths of time. Significant differences are labeled as * (*p* < 0.05).

Denture 3D+ possessed a higher DBC rate than those of the TC10 groups, probably because of the different compositions. According to its safety data sheet, NextDent’s Denture 3D+ primarily comprises monomeric components, including ethoxylated bisphenol A dimethacrylate (a difunctional monomer containing two C=C double bonds, ≥75%) and urethane dimethacrylate (another difunctional monomer, approximately 10–20%). Additionally, the formulation contains a trace (approximately 5–10%) of 2-hydroxyethyl methacrylate (HEMA), a monofunctional monomer used as a reactive diluent. All these components are methacrylate-based monomers. The formulation also incorporates approximately 5–10% of inert fillers (fumed silica), 1–5% of a photoinitiator (TPO), and traces of pigment (TiO_2_ < 0.1%). In contrast, the TC10 resin series was formulated using 90% of a rigid difunctional TCDDMDA monomer and 10% of HBPUA, containing a highly branched multifunctional methacrylate. This composition prominently increased the effective functional group density compared to that of Denture 3D+, indicating more C=C double bonds were available for cross-linking.

In the cured TC10 resins, the higher cross-linking density generated a more tightly packed polymer network. However, in the cured Denture 3D+ resin, inert fillers and monofunctional monomers diluted the concentration of reactive double bonds per unit of volume, decreasing the average number of functional groups per monomer molecule. In photopolymerized systems, higher functional group densities can reduce the final conversion degree. In early polymerization stages, highly functionalized monomers rapidly form cross-linked networks, leading to gelation and sharply increasing the systemic viscosity. In later curing stages, gelation restricted the polymer chain mobility, hindering further conversion of residual double bonds.^37^

### 3.5 Three-Point Bending Strengths, Impact Strengths, and Microhardness

Figure 7a,b shows the representative stress–strain curves of Groups 2, 4, and 7 after water storage for 50 h or 28 d, respectively. According to the ISO 20795-1 standard for denture base polymers, the flexural strength must not be below 65 MPa. In all the groups, the flexural strengths were above 65 MPa, regardless of water storage for 50 h or 28 d (Table 4). Group 4 possessed the highest flexural strength, even after 28 d of water immersion. In addition, flexural strengths usually decreased after 28 d compared with 50 h of water immersion (Figure 7c).

**Table 4:** Flexural Properties of Denture 3D+ and TC10 Resins after Water Storage for 50 h (as per the ISO 20795-1 Standard) and 28 d^a^

	Flexural strength (MPa)	Flexural modulus (GPa)	Toughness (N/mm^2^)	Elongation (%)	Flexural strength (MPa), 28 d	Flexural modulus (GPa), 28 d	Toughness (N/mm^2^), 28 d	Elongation (%), 28 d
ISO 20795-1	≥65	≥2			≥65	≥2		
Denture 3D+, 5 s	92.97 ± 1.56	2.10 ± 0.02	1930 ± 305	29.9 ± 3.30	83.71 ± 5.53	2.14 ± 0.05	829 ± 168	17.67 ± 1.98
Denture 3D+, 15 s	96.49 ± 1.43	2.02 ± 0.03	2598 ± 435	36.9 ± 4.68	90.15 ± 1.63	2.17 ± 0.05	1581 ± 350	26.15 ± 3.92
TC10-0.01, 5 s	86.41 ± 6.13	2.75 ± 0.09	565 ± 84	12.3 ± 0.94	88.71 ± 5.77	2.77 ± 0.02	591 ± 94	12.95 ± 1.06
TC10-0.01, 15 s	101.50 ± 5.30	2.65 ± 0.02	1014 ± 217	17.7 ± 2.23	91.38 ± 6.97	2.93 ± 0.04	595 ± 150	12.64 ± 1.59
TC10-0.01, 30 s	98.43 ± 5.90	2.99 ± 0.04	753 ± 123	14.4 ± 1.27	81.17 ± 9.48	2.96 ± 0.07	462 ± 112	11.44 ± 1.15
TC10-0.02, 5 s	91.95 ± 4.92	2.49 ± 0.03	762 ± 122	15.4 ± 1.32	79.70 ± 7.70	2.80 ± 0.02	455 ± 103	11.19 ± 1.25
TC10-0.02, 15 s	95.02 ± 5.13	2.68 ± 0.03	813 ± 135	16.0 ± 1.53	74.47 ± 8.65	2.92 ± 0.06	370 ± 90	9.89 ± 1.18
TC10-0.02, 30 s	90.41 ± 5.77	2.72 ± 0.04	609 ± 80	13.1 ± 0.78	90.27 ± 10.46	2.95 ± 0.04	604 ± 192	12.63 ± 2.05

a Measurements included flexural strength and modulus, toughness, and elongation at break.

**Figure 7:**
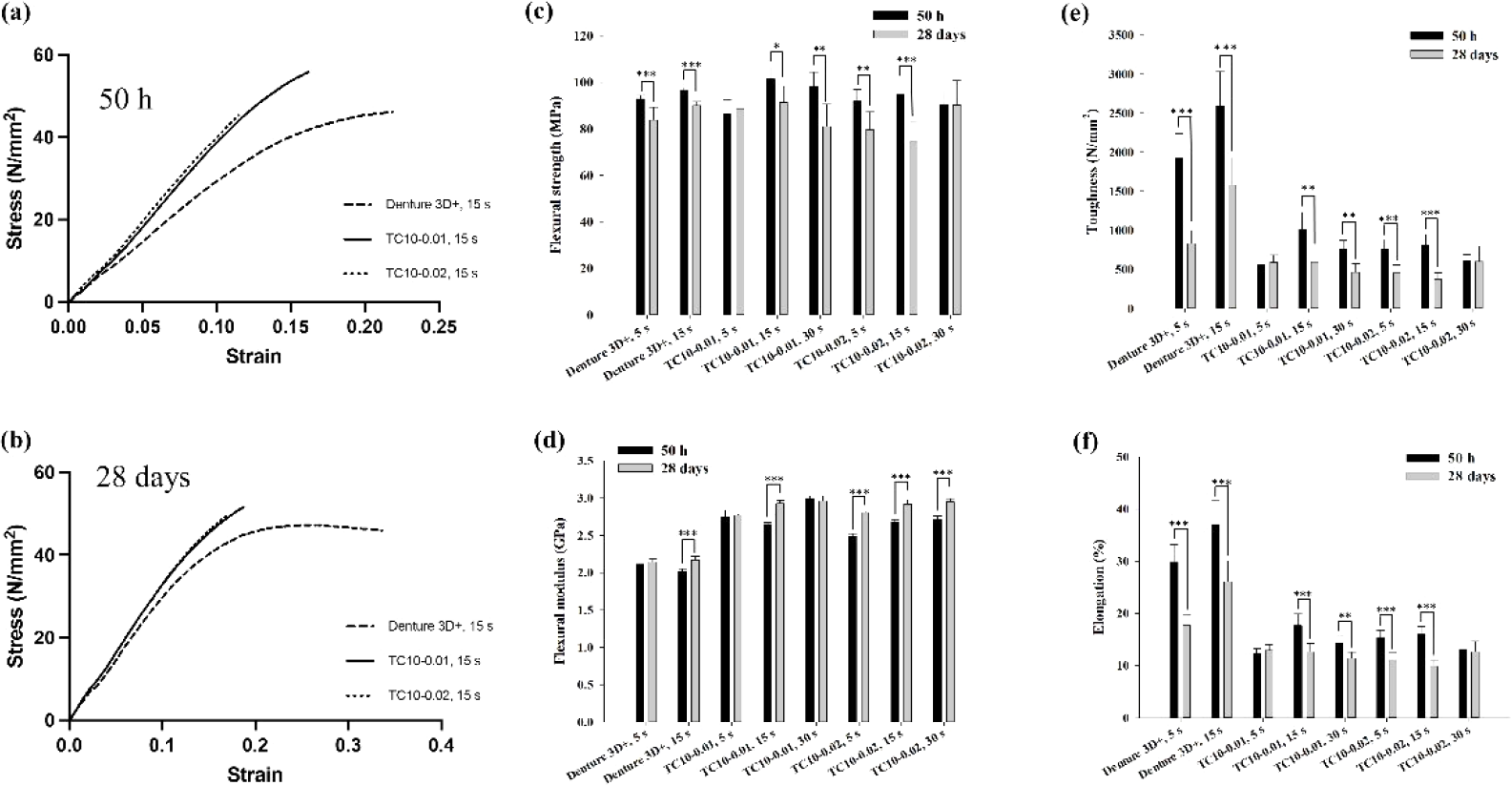
Representative stress–strain curves of Groups 2, 4, and 7 after water storage for (a) 50 h or (b) 28 d. Comparison of (c) flexural strengths and (d) moduli, (e) toughness values, and (f) elongations of different resin groups after short- and long-term water immersions (50 h and 28 d), respectively. All the tests were conducted according to the ISO 20795-1 standard. Values are expressed as means ± standard deviations. Asterisks (*) indicate statistically significant differences between 50 h and 28 d water immersions within the same group (*p < 0.05, **p < 0.01, ***p < 0.001).

According to the ISO 20795-1 standard, the flexural modulus shall be at least 2 GPa. In all the groups, the flexural moduli were above 2 GPa, regardless of water storage for 50 h or 28 d (Table 4). In addition, the flexural moduli of all the synthesized materials were higher than that of the commercial product Denture 3D+ for both water storage durations. In contrast to the flexural strengths, all the groups usually possessed increased flexural moduli after 28 d compared with 50 h of water immersion (Figure 7d).

The ISO 20795-1 standard does not regulate the toughness and elongation of denture base polymers. Groups 1 and 2 (both containing Denture 3D+) possessed higher toughness and elongation values than those of the synthesized materials in the remaining groups for both water storage durations (Table 4). In addition, in all the groups, the toughness and elongation usually decreased after 28 d compared with 50 h of water immersion (Figure 7e and f, respectively).

The impact strength of Group 2 was the highest, which was significantly greater than those of Groups 3, 6, and 7 (Figure 8a). However, no significant difference was found between Group 2 and Group 4. For microhardness measurement, Group 8 exhibited the highest value, which was significantly higher than those of Groups 2–5 (Figure 8b). In addition, no significant difference was found among Groups 2–7.

**Figure 8:**
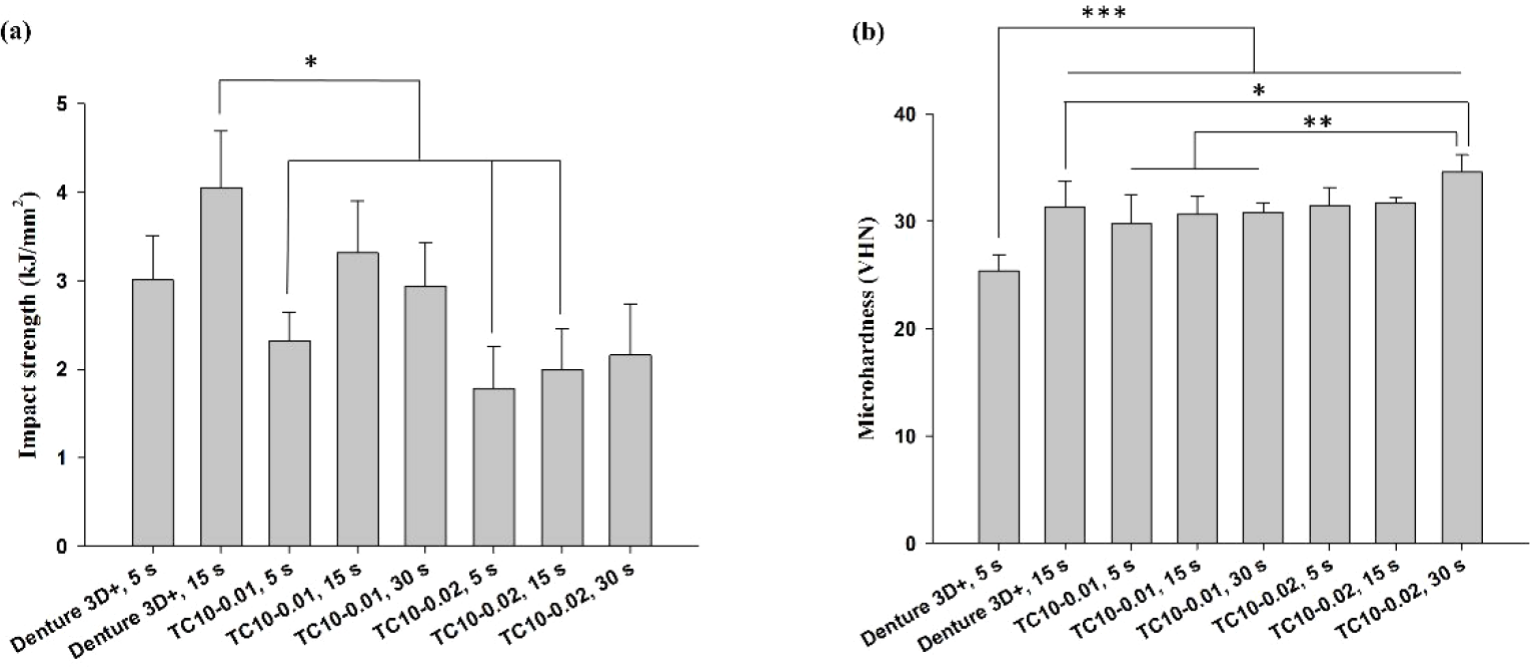
(a) Impact strength (kJ/mm^2^) and (b) microhardness (VHN) of Denture 3D+ and TC10 resins. Significant differences are labeled as *p < 0.05, **p < 0.01, ***p < 0.001.

TCDDMDA is a highly cross-linkable, rigid, bicyclic, difunctional monomer, while the proportion of flexible segments in the synthesized HBPUA was relatively low, generating an overall rigid network structure. Therefore, the TC10 materials possessed significantly higher flexural moduli than that of Denture 3D+, indicating that TC10 materials were more rigid than Denture 3D+.

Although cross-linked network structures usually stiffen materials, when the cross-linking density is too high, the glass transition temperature (*T*_g_) and chain mobility increases and decreases, respectively, contributing to increased brittleness and reduced elongation at break. Toughness, which is defined as the area under the stress–strain curve, highly depends on the material’s deformability. Poor-elongation materials fracture more readily, thereby reducing the overall toughness. These structural characteristics help to explain the lower toughness and elongation of the TC10 materials compared to those of Denture 3D+. Additionally, Denture 3D+ contains silica nanofillers. Previous studies have revealed that the incorporation of nanofillers, such as silica, can improve the monomer component distribution and reduce the number of voids within the material, enhancing intermolecular adhesion, facilitating stress distribution, and suppressing crack propagation, ultimately significantly improving both toughness and elongation.^38^

The flexural fatigue-induced fracture of removable dentures is a common cause of failure, and flexural strength, representing the maximal bending stress that a material can withstand before fracturing in a three-point bending test, is the most frequently evaluated mechanical property of denture base resins.^2^ Toughness refers to the ability of dentures to resist stress and Izod impact test indicates the energy required to break notched specimens under standard conditions. The long-term use of dentures concentrates stress at microcracks within the denture base resin, and repeated chewing force propagates cracks, ultimately causing fractures.^39^ The results of this study revealed that the TCDDMDA-HBPUA blended polymer possessed both high flexural strength and a high flexural modulus. TCDDMDA’s bulky tricyclic structure limited the polymer chain movement and increased the cross-linking density. As described previously, HBPUA contains several reactive moieties, including a poly­(ethylene oxide) structure and C=O and N–H functional groups. The TCDDMDA-HBPUA blended polymer might further reduce residual unpolymerized monomers and increase the polymerization degree.

In this study, LCD was used for 3D printing, and both the selected parameters and postcuring were crucial to produce a resin possessing the desired mechanical strength. The 30 μm high printing layer, optimal light exposure of each layer, and postcuring prevented weak adhesion between successive resin layers, which is a common cause of poor mechanical strength in 3D-printed materials.^40^

### 3.6 Surface Roughness, Shrinkage, Water Sorption, and Solubility Measurements

The surface roughness of all the TC10 resins was significantly lower than that of Denture 3D+ (Figure 9a). For the synthesized materials, shrinkage was significantly lower than that of Denture 3D+ (Figure 9b). In addition, for both TC10-0.01 and TC10-0.02, shrinkage increased with prolonged light exposure. However, for the same exposure times, no significant difference was found between TC10-0.01 and TC10-0.02.

**Figure 9:**
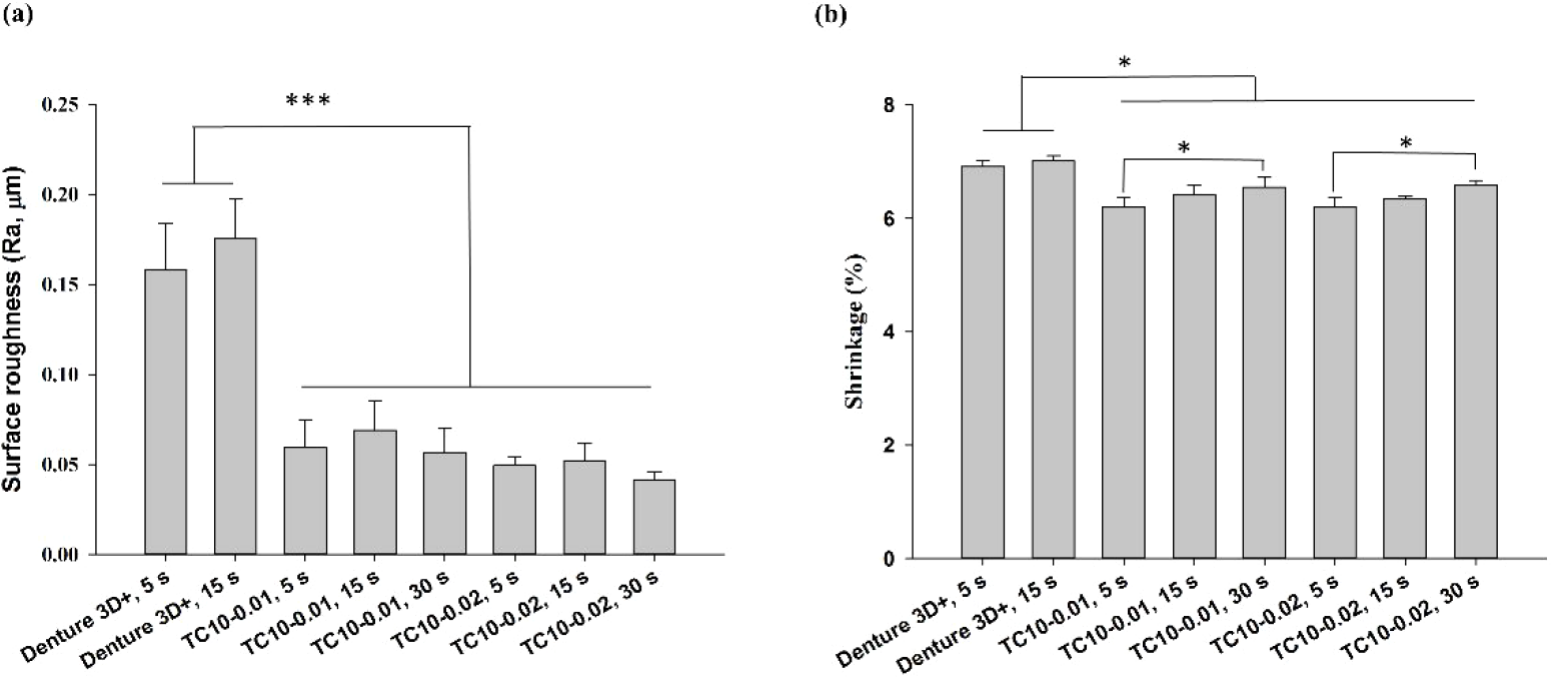
(a) Surface roughness (Ra, μm) and (b) shrinkage (%, determined by volume displacement according to Archimedes’ principle). Significant differences are labeled as *p < 0.05, ***p < 0.001.

Figure 10a–h displays photographs of representative printouts of Groups 1–8 for water sorption and solubility measurements, respectively. The ISO 20795-1 standard suggests that the water sorption and solubility should be ≤32 and ≤1.6 μg mm^–3^, respectively. The results revealed that in all the groups, the solubility in 37 °C water for 5 d was below 0.1 μg mm^–3^. The water sorption of the synthesized materials ranged from 7.98 ± 0.38 to 8.84 ± 0.43 μg mm^–3^ (for TC10-0.02, 30 s and TC10-0.01, 5 s, respectively). The values in these groups not only met the ISO 20795-1 regulation but also were significantly lower than that of Denture 3D+ (Figure 10i).

**Figure 10:**
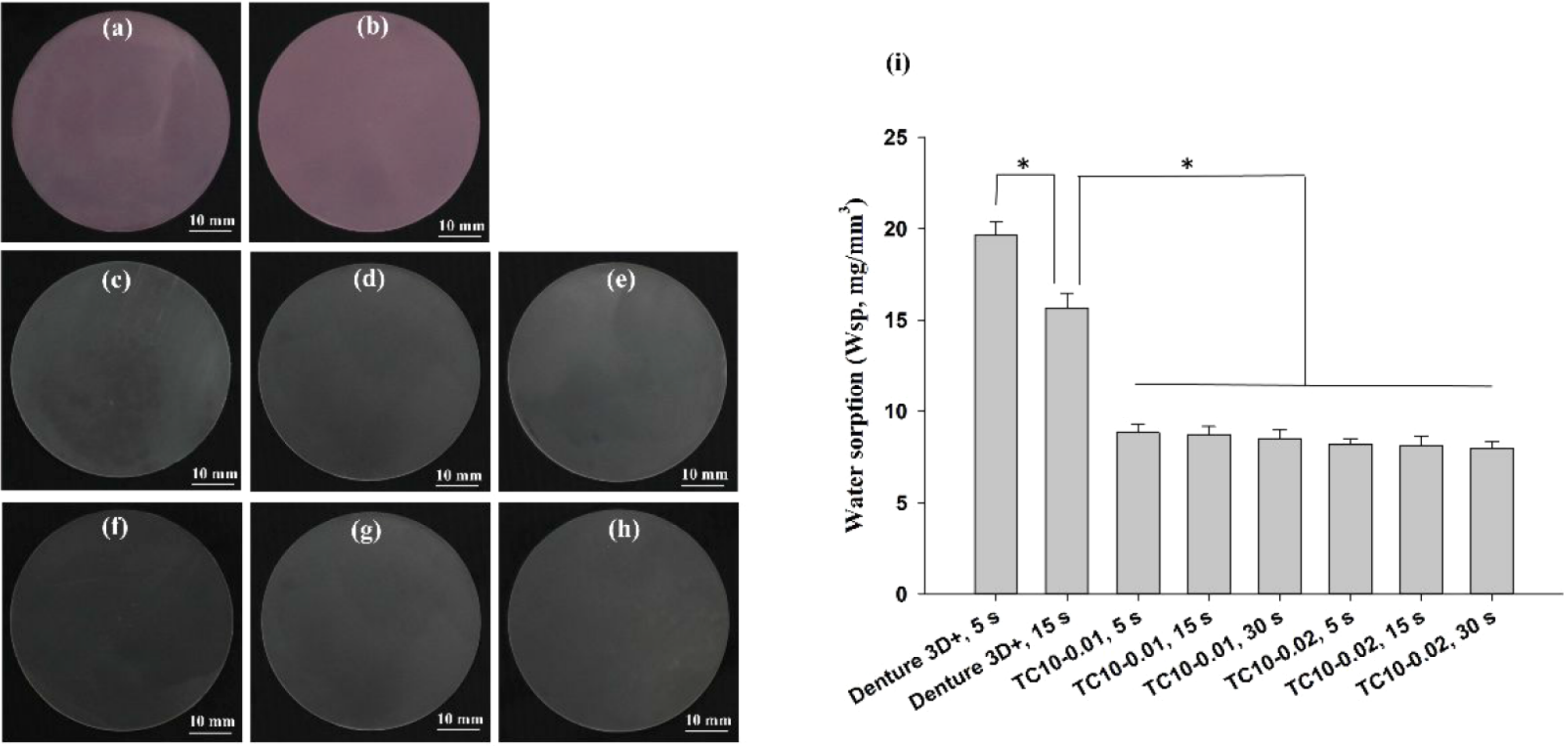
(a–h) Photographs of representative printouts of Groups 1–8, respectively. (i) Water sorption values (*W*sp, mg mm^–3^) of Denture 3D+ and TC10 resins, as determined according to the ISO 20795-1 standard. Significant differences are labeled as * (*p* < 0.05).

Water sorption is the amount of water absorbed by materials, which can impair their mechanical properties and thermal stability.^41^ Water solubility represents the number of unreacted monomers and hydrolysis degree. Therefore, both properties should be as low as possible for long-term use of denture base resins. Denture 3D+ contained a higher proportion of long-chain polymers and hydrophilic functional groups (HEMA) than those in the TC10 resins, which increased the material’s moisture affinity, significantly increasing the water sorption compared to those of the TC10 resins. In contrast, in the TC10 resins, TCDDMDA was a hydrophobic cross-linking monomer, and a high cross-linking degree could reduce water sorption and solubility. The contact angles of Denture 3D+ and Group 7 were 68.6 ± 0.4 and 78.6 ± 0.3, respectively, indicating that TC10 resins were more hydrophobic than Denture 3D+ and, consequently, possessed significantly lower water sorption rates.

### 3.7 Material Biocompatibilities

For all three experimental materials, CCK-8 assays revealed that the cell viabilities (%) slightly decreased with prolonged incubation (Figure 11). The ISO 10993–5 standard regulates that a reduction in cell viability by more than 30% is a cytotoxic effect; therefore, as all the cell viabilities exceeded 70%, all the materials were noncytotoxic. In addition, although TC10-0.01 possessed approximately the same cell viability as Denture 3D+, TC10-0.02 possessed the lowest cell viability.

**Figure 11:**
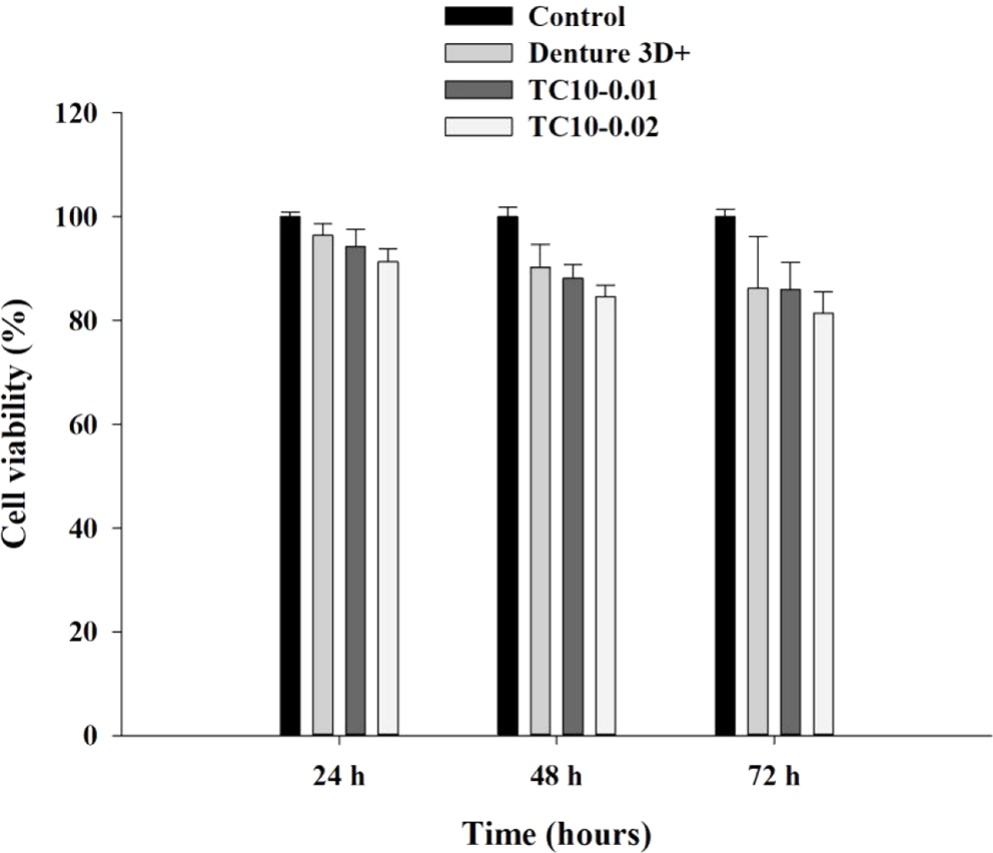
HGF-1 cell viabilities for Denture 3D+, TC10-0.01, and TC10-0.02, as determined using a CCK-8 assay.

TCDDMDA-denture base resin copolymers have previously been shown to be noncytotoxic to murine fibroblasts.^30,31^ Moreover, a previous study also revealed that HBPUA possessed biocompatibility similar to that of the control.^27^ In addition to the inherent biocompatibilities of TCDDMDA and HBPUA, cell viability is also influenced by the unreacted residual monomer content in specimens. The CCK-8 assays revealed that the TCDDMDA-HBPUA blended polymer did not release any harmful unreacted residual monomers into the cells.

## 4 Conclusions

The fracture of conventional PMMA-based resins is a common cause of denture base failure, and conventional fabrication using traditional heat-induced polymerization is time-consuming. In this study, we developed a denture base material comprising 90 and 10 wt % of TCDDMDA and HBPUA, respectively, using LCD 3D printing. The results showed that Group 4 possessed the highest flexural strengths after 50 h and 28 d of water immersion. The flexural modulus of Group 4 not only satisfied the ISO 20795-1 requirement but also exceeded the flexural modulus of Denture 3D+, although the toughness and elongation of Group 4 were lower than those of Denture 3D+. In addition, the impact strength and microhardness of Group 4 were comparable to those of Denture 3D+. Furthermore, the water sorption and solubility of Group 4 were far below the ISO 20795-1 requirements and superior to those of Denture 3D+. Group 4 also exhibited low surface roughness and volumetric shrinkage. CCK-8 assays revealed that Group 4 possessed the same high biocompatibility as Denture 3D+. These results revealed that although Group 4 has good potential as a denture base material, further clinical study is required to prove its efficacy.

## Data Availability

The data underlying this study are available in the published article.
